# Supplementary material for: Are carbon emissions trading and green financial instruments synergistic? -Comprehensive quantitative research based on content analysis
Source: PLoS One. 2024 Mar 7;19(3):e0298601. doi: 10.1371/journal.pone.0298601 (PMC10919601; doi:10.1371/journal.pone.0298601)
Supplement: S1 Data — (ZIP) [file pone.0298601.s001.zip › Data/地级市Z_F工作报告.zip!/░▓╗╒/┴∙░▓╩╨/└┤╫╘╣1⁄2╓┌║┼í╛┬φ┐╦╩2╛▌═°í┐_2009.docx]

各位代表：
　　现在，我代表市人民政府，向大会作政府工作报告，请予审议，并请市政协委员和其他列席人员提出意见。

**一、攻坚克难，2008年经济社会发展跨上新台阶**

　　2008年，是我国改革发展进程中非常重要的一年，也是非同寻常的一年。一年来，全市上下在中共六安市委的坚强领导下，认真贯彻党的十七大和胡总书记两次视察安徽重要讲话精神，以科学发展观为统领，解放思想，团结拼搏，圆满完成了市二届人大四次会议确定的目标任务。
　　全年生产总值达534亿元，增长13.8%；人均生产总值8754元，比上年增加1532元。三次产业结构由上年的25.5：36.1：38.4调整为24.2：38.6：37.2。财政收入42.3亿元，增长28.5%。固定资产投资256.2亿元，增长27.7%。城镇居民人均可支配收入11807元，农民人均纯收入3647元，分别增长12.9%和19.3%。国民经济保持了速度较快、效益较好、结构优化、民生改善的良好态势，社会事业全面发展，社会大局和谐稳定，综合实力跨上新台阶。
　　第一，坚持工业化核心战略，切实加快工业发展步伐。先后召开工业学霍山现场会、金寨汇报会、开发园区建设现场会，采取一系列鼓励扶持措施，强力推动工业经济快速发展。规模工业增加值143.5亿元，增长27.4%，总量在全省前移2位；新增规模企业133家，总数达687家；亿元企业达106家；效益综合指数201.3，比上年提高21.5个百分点。认定国家级高新技术企业22家，高新技术产业产值达44.2亿元，增长43.1%，增幅居全省第5位。编制重点产业发展规划，推进钢铁双百亿工程，钢铁产业产值达80亿元。着力加强项目建设，工业固定资产投资105.2亿元，增长45.1%；应流合金钢坯、迎驾溢彩玻璃器皿、雪花啤酒一期、星瑞乘用车变速箱、江淮节能电机扩建、金安不锈钢合金钢一期、鸿福晶体二期、索伊冰箱扩建、金安矿业300万吨选矿、江淮永达等一批重大项目建成；华电六安电厂二期项目已经国家批准开展前期工作，大昌100万吨球墨铸造项目通过环保部环评。组建了市工业投资公司，稳步推进恒大集团、华源纺织等企业改革。大力实施名牌战略，长江精工钢结构件成功入选中国名牌评价目录，新增国家地理标志保护产品2个、安徽名牌产品8个。园区建设取得新进展，9个省级开发园区新增建成面积9平方公里，完成基础设施投入9亿元。强力推进节能减排，单位生产总值能耗下降7%，五县污水处理厂全部建成，污染减排圆满完成省下达的目标任务。
　　第二，深入实施招商引资主战略，不断扩大对外开放。积极参与泛长三角区域合作，认真实施《安徽省会经济圈规划纲要》，积极加强对接。突出领导带头招商，市领导带队外出招商25次；调整充实市直23支招商小组，设立6个驻外办事处，新组建中央、省属驻六安单位21支招商小组和6个在外人才协会分会；修订了优惠政策，出台了社会招商奖励办法；积极参加第三届中博会、徽商大会等商贸活动。招商到位内资132.6亿元、外资1.1亿美元，分别增长36.8%、44.9%。采取出口补贴和信用补贴等措施，进出口总额达3.3亿美元，增长33.3%。更加注重亲商安商，在全市设立145个投资环境监测点，出台《行政机关及其工作人员损害经济发展环境处理暂行办法》等措施；全市共受理投诉案件212件并全部办结，纠退资金345.7万元，避免企业损失3652.8万元；开展行政审批项目第三轮清理，取消和调整82项。
　　第三，积极发展现代农业，扎实推进新农村建设。认真贯彻十七届三中全会精神，不断推进农村改革与发展。加强耕地保护，促进适度规模经营，多种形式的土地流转面积达114万亩，全市种养业经营土地百亩以上大户816户、千亩以上37户。强力推进现代农业十大基地建设，启动粮食双百亿工程，全面开展农业政策性保险。粮食总产达437.4万吨，连续四年创历史新高，霍邱县连续四年荣获全国粮食生产先进县标兵称号，寿县再次荣获全国粮食生产先进县称号。实施农业产业化倍增计划，新增国家级龙头企业1家、省级12家、市级77家。有机、绿色、无公害农产品认证总数达160个，认证面积200多万亩，居全省前列。推进科技兴农，选派科技特派员402名，创建专家大院41家。全面实施惠民直达工程，累计发放各类惠农补贴16.4亿元。全国农村公路示范区建设进展顺利，完成村村通投资4.45亿元，建成2077公里。治淮14项骨干工程全面完成，农业综合开发和江淮分水岭开发继续推进，农田水利基本建设和土地整理成效显著，60座小型病险水库除险加固全面开工，解决了37万人饮水安全问题。加强重点地质灾害危险点搬迁避让和工程治理，全面完成14处省级和26处市级地质灾害点治理工作。农村综合改革、集体林权制度改革和水管体制改革积极推进。一县两区被省列入土地利用规划修编试点，第二次土地调查扎实开展。
　　第四，强力推动中心城市和重要基础设施建设，努力加快城镇化进程。中心城市完成政府性投资11.7亿元，建成区面积达51.3平方公里，人口50万人；完成中心城市新一轮总规评估，制定重点工程三年实施计划，完成15亿元城投债券发行的各项准备工作，随时可择机上市；实施淠河综合治理，橡胶坝中坝等工程顺利开工，开启了中心城市建设新篇章；坚持重心下移，改革完善了土地征用、拆迁安置办法；完成合六叶高速连接线、淠河总干渠景观带（和顺段）等60项年度重点工程建设任务，旧城改造加快推进；全面实施绿化、美化、亮化工程，六安被命名为省级园林城市；加大投入，中心城市数字化管理工作上了一个新台阶。基础设施建设快速推进，合武铁路建成运营，合淮阜高速公路开通，合武、六潜高速公路六安段加快施工；500千伏输变电工程建成运行；内河整治、大型泵站更新改造、洼地治理和淠史杭灌区配套与节水改造顺利实施。县城和50个重点镇建设取得进展，城镇化率达35.4%。
　　第五，大力发展服务业，努力扩大消费需求。实现社会消费品零售总额198.2亿元，增长23.1%。光彩大市场、百大金商都等重点项目建成运营。积极巩固万村千乡市场工程建设成果，加快推进新网工程，县及县以下消费品零售总额增长18.3%。旅游发展势头较好，实现总收入25亿元，增长25%。认真实施旅游发展三年行动计划，旅游基础设施和接待条件得到改善。红色旅游规划编制完成，旅游总体规划修编顺利。金融保险等现代服务业加快发展，金融存款余额539.7亿元，比年初增加114.47亿元；贷款余额273.58亿元，比年初增加51.07亿元、居全省第3位；积极开展银企对接，落实意向授信额度106亿元；金融生态明显改善，贷款不良率下降16.32个百分点，金融机构盈利5.84亿元，是上年的1.92倍。保险业持续发展，实现保费收入23.2亿元。
　　第六，高度关注民生问题，着力构建和谐六安。18项民生工程全面实施，工作扎实、成效显著，在全省率先建立民生工程预算制度，资金总投入20.1亿元，其中市县配套4.6亿元，惠及510多万城乡居民，在全省年度考评中荣获第一名。全面落实再就业政策，新增就业34584人，登记失业率控制为4%，实现再就业15799人，其中困难人员3382人。继续扩大社会保险覆盖面，连续四年调高企业退休人员养老金。注重保护被拆迁人利益，建成安置房20.4万平方米，发放廉租住房补贴8987户、1137万元，实物配租385户。加大扶贫开发力度，整村推进185个村、脱贫8.8万人。发放库区移民直补及项目扶持资金4.8亿元。各级各类教育健康发展，教育质量不断提升，义务教育经费保障机制改革全面实施，城区义务教育学校改扩建工程继续推进，完成D类危房改造37万平方米。加快城乡卫生服务体系建设，市医疗紧急救援中心正式启用，完成29个社区卫生服务机构规范化建设，改建乡镇卫生院45个、村卫生室290个；新农合覆盖面不断扩大，参合农民490万人，参合率达89.7%。推进农村电影2131工程、送戏下乡、农家书屋建设，4400多个村开通广播电视；广泛开展群众文化活动，成功举办中国大别山读书节、中国大别山民歌会等活动。大力实施人才强市战略，各类人才队伍建设得到加强。市二运会成功举办，全民健身中心启用，群众性体育运动蓬勃开展。人口和计划生育 “七到位两规范”工作扎实推进，低生育水平保持稳定，人口出生率控制在12.3‰以内。计划生育设施建设步伐加快，新改建乡镇服务所67个、村服务室1447个，霍山县被评为全国计划生育优质服务先进县。坚持领导信访接待日制度，扎实开展矛盾纠纷排查化解和重信重访专项治理活动。安全生产形势明显好转。社会治安综合治理全面推进，公安三基工程建设基本完成，人民群众安全感和满意度明显提升。依法加强宗教事务管理，五五普法和依法治市工作深入推进。强化食品药品安全监管，开展百日联合行动等专项整治，市农产品检测中心挂牌运行。关心驻地部队和武警部队建设，国防动员、民兵预备役、人防和双拥工作进一步加强。妇女、儿童、老龄、残疾人事业取得新进展。
　　各位代表，面对年初罕见的特大雪灾，我们积极发动，迅速应对。全市30万干群、万余台机器战斗在道路一线，扫雪除冰、抢通保畅，境内高速公路、国省道和重要县道总体通畅，无滞留人员；紧急做好受灾群众转移和安置工作，迅速下拨救灾款物，应急救灾工作受到国家有关部委和省领导充分肯定；灾后重建成效明显，按时按质完成倒房重建任务。果断应对突如其来的手足口病疫情并取得重大胜利。全市人民心系灾区，积极支持四川抗震救灾和灾后重建，募集各类资金6108万元，圆满完成医疗救护和援建都江堰市幼儿园及外国语实验学校校舍任务。积极应对和妥善处置问题奶粉事件，及时筛查婴幼儿15万人次，全力免费救治患儿，切实开展问题奶粉市场清查和监管。一系列重大突发事件的有效应对，保障了人民群众的生命财产安全，维护了社会稳定。
　　面对宏观形势的急剧变化，我们积极贯彻落实中央和省促进经济平稳较快发展的各项政策，行动迅速，措施有力，当即召开投资和项目工作会议进行部署，出台促进经济平稳较快增长20条意见，派出206个工作组进驻企业，成立4家小额贷款公司。据统计，全市共安排工业发展专项资金7400万元，担保机构增资2.4亿元，中小企业贷款风险补偿资金规模达6800万元。在国家新增1000亿元国债投资中，共争取到补助项目74个、补助资金6.5亿元，资金额占全省1/7。通过采取一系列卓有成效的非常措施，保持了经济平稳较快发展的良好势头。
　　在推进经济社会发展的过程中，我们高度重视政府自身建设，努力建设勤政、廉洁、务实、高效政府。共办理人大议案3件、代表建议90件和政协委员提案215件。推进科学民主决策，修订了市政府工作规则，完善专家参与重大决策机制，续聘市政府法律顾问和行政执法监督员，加强行政复议和行政应诉工作，举办市政府法制讲座2次。认真开展反腐倡廉制度建设推进年活动，市直清理制度2156项、新建和修订完善822项。扩大政务公开渠道和范围，启动政府信息公开，市政府网站连续4次进入全国50强。推进应急管理体系建设，应对突发事件能力得到增强。加强廉政建设，强化行政监察、审计监督和招投标监督管理，加大对商业贿赂和损害群众利益不正之风的治理力度，政风行风建设取得新成效。
　　各位代表！回顾过去的一年，发展令人振奋，成绩来之不易。这是省委、省政府和中共六安市委正确领导的结果，是全市人民团结拼搏、开拓奋进的结果。在此，我谨代表市人民政府，向全市人民，向在我市创业的投资者、建设者们，向所有关心、支持六安现代化建设的朋友们，致以崇高的敬意和衷心的感谢!
　　我们清醒地看到，我市经济社会发展中还存在许多困难和问题。又好又快发展的基础不够稳固，工业化水平较低，新兴产业发展不快，非公经济发育不足，中小企业活力不强，经济增长的资源环境代价较大；农业基础仍然薄弱，农民持续稳定增收的压力加大，统筹城乡发展任务艰巨；部分群众生活比较困难，就业再就业形势严峻，社会保障体系有待完善；政府职能转变还不完全适应改革发展要求，行政效能仍有待提高。对此，我们一定高度重视，采取有效措施，认真加以解决。

**二、坚定信心，扎实做好2009年各项工作**

　　2009年可能是近年来经济发展最为困难的一年，也是蕴含重大机遇的一年。如何积极有效地应对宏观环境变化的影响，千方百计保持良好发展势头，是最重要的任务，也是我们面临的最大挑战。应当看到，金融危机的影响还在持续和蔓延，随着危机影响滞后效应的进一步显现，发展形势将更加严峻。但更要看到，六安作为经济外向度较低的后发地区，危机影响相对较弱，范围相对较小；六安多年发展能量的积聚和释放，将为经济注入长久的发展动力；特别是国家实施积极的财政政策和适度宽松的货币政策，已经出台和正在谋划一系列促进经济增长的激励措施，将给发展带来新的机遇。总体上看，支撑我市经济发展的中长期因素没有改变，厚积薄发、加速崛起的态势没有改变，全市上下加快发展的强烈愿望没有改变。因此，面对困难，我们既要看到形势的严峻性，增强忧患意识；应对挑战，我们更要看到潜在的能量，增强机遇意识，坚定信心，克难而进，加快发展，奋力崛起。
　　根据市委二届十一次全会的部署，今年政府工作的总体要求是：以邓小平理论和三个代表重要思想为指导，以科学发展观为统领，坚持工业化核心战略、招商引资主战略和城镇化带动战略不动摇，以保持经济社会平稳较快发展为首要任务，扩大有效投入，调整经济结构，深化改革开放，保障改善民生，全面完成市第二次党代会和二届人大一次会议确定的目标任务，为实现六安由农业大市向农业强市、工业小市向工业大市、财政穷市向财政富市的新跨越奠定坚实基础。
　　综合考虑各种因素，今年经济社会发展主要预期目标是：生产总值增长12%，财政收入增长20%，规模工业增加值增长20%以上，全社会固定资产投资增长30%，社会消费品零售总额增长15%，进出口总额增长15%，招商到位内资和外资均增长15%以上，城镇居民人均可支配收入和农民人均纯收入分别增长10%、8%，居民消费价格涨幅控制在4%左右，城镇新增就业2.64万人以上，城镇登记失业率控制在4.2%以内，人口出生率控制在13.7‰以内，单位生产总值能耗下降7%，化学需氧量和二氧化硫排放量分别下降4.53%、5.62%。
　　（一）着力引导投资增长，努力扩大消费需求。紧紧抓住国家加大投入、扩大内需的有利机遇，迅速有效地拉动投资增长，积极主动地扩大消费需求，强化经济增长的支撑力。
　　加强基础设施建设。积极做好高速公路和铁路建设的协调服务，力争六潜高速建成通车、六武高速年底竣工，争取阜六高速尽快复工；支持合武铁路正式运行和宁西铁路复线、阜六铁路开工建设，做好六安至庐江和六安至安庆段铁路建设的前期工作。推进国道、省道及重要县道改造；加强农村公路建设，建成村村通1800公里，改建通乡油路380公里。认真实施《六安港总体规划》，支持新桥国际机场建设。推进电力、通信等基础设施建设，新建一批220千伏、110千伏输变电等工程。
　　推进产业项目建设。力促华电60万千瓦、大昌100万吨球墨铸造、众兴集团矿石采选、应流特种大型铸件、金安不锈钢深加工、蓝翔玻璃等项目尽快开工，加快粮食物流园区、长江彩铝、鸿阳新能源、海螺水泥粉磨站、博微电子、财富特种车、华源制药、万佛湖旅游综合开发、红色旅游开发等项目建设，确保3000万元以上重大产业项目投资达180亿元。同时，紧紧围绕国家重点投资领域，谋划一批牵动性强的重大项目。
　　加快融资体系建设。贯彻落实适度宽松的货币政策，综合运用担保、贴息、风险补偿等措施，引导金融机构加大信贷投放，在加大银企对接、力争新增贷款60亿元的同时，注重调整贷款结构，加大工业贷款投入。鼓励适合中小企业的金融创新，努力解决中小企业融资难问题。积极推动农村金融产品和服务方式创新，加快推进小额贷款公司、村镇银行和农村资金互助社等新型金融组织试点推广工作，支持符合条件的农村信用社组建农村合作银行或农村商业银行，争取市外金融机构来我市开展信贷业务和设立分支机构。积极推进企业上市和境外融资。促进保险业健康发展，充分发挥保险业风险补偿作用。
　　扩大消费需求。加快市县商业网点规划和实施，推进商贸流通双百工程。以拓展农村市场为重点，抓好万村千乡市场工程固点扩面，推进新网工程，组织开展家电下乡等活动，支持大型商业连锁企业向农村延伸网点。加快发展城市商贸服务业，健全城市社区便民服务网络，规范发展旧货回收和再生资源利用。大力发展旅游业，完成全市旅游总体规划修编，继续推进旅游发展三年行动计划，抓好重点旅游项目建设，着力提升景区品质；进一步打造以原生态和红色旅游为品牌的大别山旅游和以寿县古城为龙头的淮河文化风情旅游，发展生态游、乡村游、农家乐等特色旅游。支持大别山（六安）国家地质公园争创世界地质公园，办好立夏节起义、六霍起义胜利80周年庆祝活动。
　　（二）坚持工业化核心战略不动摇，全面提升工业经济水平。进一步深化“发展工业学霍山”活动，着力化解各种不利因素，积极推进产业升级和自主创新，全力以赴保企业、保增长，确保工业固定资产投资增长25%以上。
　　做大工业经济总量。按照增特色、壮龙头、促配套、可持续的原则，推动产业结构优化升级。围绕矿产冶金、机械制造、纺织服装、能源和农副产品深加工等产业龙头企业及产业链延伸，组织实施100个投资超千万元项目，培育超亿元企业120家以上。强力推进钢铁双百亿工程，力争钢铁产业产值达100亿元以上。积极培育产业集群，加快壮大省认定产业集群专业镇的集聚规模，加强光伏材料、机械制造等产业规划与引导，逐步形成较为鲜明的产业集群构架。
　　增强企业创新能力。利用市场约束的倒逼机制和经济调整的洗牌效应，大力发展高新技术产业，着力增强企业创新能力，积极扶持创业辅导、风险投资、技术开发等各类服务机构建设。大力促进产学研合作和科技成果转化，重点推进30个技术创新项目加快实施。落实科技创新政策，加大专利创造和实施力度。加快科技创业中心平台建设，努力提升入孵企业科技含量和规模。继续实施名牌战略，提升企业质量管理和标准化管理水平。
　　加快开发园区建设。健全管理体制和运行机制，进一步加快园区基础设施和项目建设。六安开发区要力争完成基础设施建设投入3亿元以上、新增建成区3平方公里以上，引进超千万元项目25个以上、到位资金15亿元以上；各县区省级开发区要力争完成基础设施投入1 亿元以上、新增建成区1平方公里以上，引进超千万元项目15个以上、到位资金6亿元以上。优先安排开发园区用地指标，引导并推行专业园区建设。开展园区土地利用状况普查清理，科学规划建设乡镇工业集中区。
　　加大对工业企业扶持力度。坚持“四个定期”、“五个倾斜”， 完善领导联系企业和项目工作制度，全面落实各项扶持政策，对涉企收费项目，采取取消、下调、暂缓征收等措施，进一步减轻企业负担。充分发挥工业投资公司平台作用，做大做强中小企业担保机构，进一步扩大各级中小企业发展专项资金和贷款风险补偿基金规模，加强煤电油运和资金的供需衔接，着力扭转部分行业和企业生产下滑的势头。强化经济运行统计分析和监测预警。
　　（三）扎实推进农村改革发展，加快新农村建设步伐。按照十七届三中全会的部署和要求，突出稳粮、增收、强基础、重民生四大重点，深化农村改革，加快农村发展。
　　大力发展现代农业。坚决落实最严格的耕地保护和节约用地制度，坚持把粮食生产放在首位，加快实施粮食双百亿工程，推进水稻产业提升行动和小麦高产攻关计划，发展农业机械化，依靠科技，提高单产，改善品质，确保粮食增产5亿斤以上。着力打造粮食产业园区，确保粮食加工产值增加10亿元以上。加快实施农业产业化倍增计划，大力发展带动力强的龙头企业，力争农产品加工产值达200亿元以上。强力推进十大基地建设，重点发展畜牧水产规模养殖，新增规模养殖小区50个。加强农产品质量安全建设，严格农产品质量安全全程监控。
　　继续加强农业基础设施建设。实施土地整治，推进高标准农田建设，改造中低产田20万亩。加快国家大型商品粮基地和寿县国家级基本农田示范区建设，实施农业综合开发土地治理10万亩。加强水利建设，支持白莲崖水库建成运行，完成正南洼地、城西湖、临王段三大泵站更新改造，完成安丰塘、龙潭、老圈行3座中型和40座小型病险水库除险加固。加快淠河、史河等主要内河及洼地治理，编制中小河流治理规划。推进淠史杭大型灌区续建配套与节水改造、江淮丘陵蓄水工程，实现38万人饮水安全。逐步健全农村公共设施维护和公益事业建设机制。
　　着力促进农民增收。认真落实惠农政策，全面推进惠民直达工程。大力实施阳光工程和百场千户万人农业科技培训，着力培训新型农民。推进农民专业合作组织建设，通过产业化带动农民加快增收。开展“神农杯”创业竞赛活动，支持返乡农民工创业，建设农民工创业园区。坚持开发式扶贫，实现农村最低生活保障制度和扶贫开发政策有效衔接，抓好国家扶贫连片开发项目试点，探索库区、深山区群众易地扶贫试点；实施整村推进180个村，脱贫8万人。切实做好农民负担监管，严格执行粮食最低收购价保护政策，加强农资等生产资料市场监管。
　　加快推进农村改革。按照依法自愿有偿的原则，探索建立农村土地承包经营权流转市场，逐步培育一批百亩、千亩乃至万亩的现代农场。创新科技服务三农长效机制，扎实推进科技特派员行动和专家大院建设。积极探索集体林权制度配套改革，完成水管体制改革。推进政策性农业保险，实施支农资金和项目整合。引导城市资金、技术、人才、管理等生产要素向农村流动，形成城镇化和新农村建设互促共进机制。抓好新农村百村十镇示范工程，科学规划和建设新农村居民点。
　　（四）推动中心城市新一轮大发展，加快城镇化进程。坚持以更高站位、更新定位、更大力度推进中心城市建设，向着120平方公里、120万人口新一轮城市总体规划远景目标奋力迈进。
　　强力推进淠河综合治理。围绕打造滨水宜居生态城市的目标，将淠河建成中心城市的生态轴、景观轴、发展轴。切实抓好河西景观大道、月亮岛环岛路、橡胶坝、淠河景观带、淠河护岸和河床整治、平桥排涝泵站、淠河截污等工程建设，统筹安排工程设计、拆迁安置、施工组织、资金筹措等环节任务，确保橡胶坝中坝4月底前竣工。切实抓好施工调度和质量监管，把淠河综合治理项目建成精品工程。
　　加强中心城市重点工程建设和管理。完成新一轮城市总规修编，编制河西新区等重要地段控制性详规。扎实推进三年实施计划，实行市领导联系重点工程制度，着重抓好淠河总干治理、中央公园、淠望路大桥、南门塔公园、西大街改造、常青路步行街、皖西大道改造、解放路改造等20项重点工程。进一步完善城市功能，注重路网管网建设，加快旧城改造和基础设施配套建设。创新城市管理模式，推进数字化城市管理信息平台建设，综合整治市容市貌，建立健全长效管理机制，提升城市管理水平。加强社区建设，切实增强综合服务功能。加强绿化和绿线管理，积极争创国家级园林城市和省级文明城市。
　　抓好县城和小城镇建设。坚持分类指导、突出特色的原则，加强规划编制和建设管理，力争建制镇控规覆盖率达70%以上。加强城镇基础设施建设，谋划一批促进城乡互动发展、推动全局发展的重大基础项目。整治市容市貌，提高城镇综合管理水平。加强社区建设，充分发挥社区服务功能。引导人口、产业向县城和中心镇集中，促进城乡相融，形成县域发展新格局，力争城镇化率提高2个百分点。
　　促进房地产业健康有序发展。提高住宅小区的建设品位和配套档次，加大廉租房、安置房等保障性住房的建设力度，发放廉租住房补贴5000户，实物配租1100户。进一步落实促进合理住房消费的政策措施，提高居民购买能力。积极开展房地产展示促销活动，加快发展二手房市场和住房租赁市场。加强物业管理，提升服务水平。
　　（五）进一步深化改革，继续扩大开放。坚持向改革要空间、要潜力、要动力，以开放促调整、促发展、促提升，以深化改革增添新动力，以扩大开放促进大发展。
　　提升开放型经济水平。在坚持各类有效招商方式的基础上，进一步推进产业集群式、产业链式和专业园区招商，围绕重点产业，摸排一批意向区域、意向企业和外商，积极引进产业关联度高、带动力强的重大项目。充分发挥招商小组、驻外联络处和在外人才协会的作用，建立驻外联络处工作考评机制，构建常态化的招商平台。进一步拓宽利用外资的渠道和领域，提升工作质量和水平。认真筹备参加第四届中博会、徽商大会等重大招商活动，切实抓好签约项目的跟踪落实。着力壮大外贸主体，做大做强羽绒、柳编、机械制造等优势产业和企业，积极调整和优化外贸出口结构。推动规模以上工业企业开展进出口备案登记，力争全年有外贸实绩企业达110家以上。继续鼓励企业“走出去”，开展对外承包工程和劳务合作。
　　积极参与泛长三角区域发展分工。加强对长三角和沿海产业转移新形势、新特点的研究，编制承接产业转移规划和方案。发挥资源、区位和部分产业的比较优势，加强合作平台建设，逐步培育一批功能明确、配套便捷的产业承接园区，在承接产业转移中推动产业升级。围绕《安徽省会经济圈发展规划纲要》，提出我市的具体实施方案，突出产业发展、基础设施、市场平台、环境保护等重点，抓好方案实施、项目谋划和工作落实。
　　发展非公有制经济。贯彻非禁即入原则，推进公平准入，促进全民创业。积极引导非公有制企业推进技术创新，引进先进管理理念和管理方法，提升企业核心竞争力。抓住沿海地区产业升级转型的机遇，促进在外人士凤还巢和回乡创业。全面落实省支持非公经济发展30条等政策措施，进一步扩大中小企业创新资金规模，切实帮助中小企业排忧解难，着力破解资金、土地、市场等难题，在全社会营造发展壮大非公有制经济的良好氛围。
　　加快改革进程。进一步推进事业单位全员聘用制，积极推动事业单位人员实现由身份管理向岗位管理转变。加快政府与市场中介组织分离，促进行业协会和中介组织向市场化、多元化、规范化方向发展。全面推进增值税转型，切实做好工商系统市场办管分离和燃油税、资源税等改革工作，继续深化企业改革。加强国有资产管理，加快预算管理制度改革，强化各类政府性资金管理，调整优化财政支出结构，加大对经济社会发展薄弱环节支持，增加改善民生支出。
　　（六）高度关注民生，加快构建和谐六安。坚持把改善民生作为一切工作的出发点和落脚点，带着责任、充满感情、千方百计把广大人民群众的利益维护好、实现好、发展好。
　　着力实施民生工程。充分发挥民生工程拉动内需、促进城乡居民共享改革发展成果的“双重效应”，在继续巩固18项民生工程成果的基础上，突出社会建设、基础建设和素质建设，将民生工程扩大至28项，新增农村五保供养机构建设、重度残疾人生活救助、新型农民培训、农民工技能培训、提高妇女儿童健康水平、农家书屋工程、乡镇综合文化站建设、农民体育健身工程、政策性农业保险、农村沼气建设工程、病险水库除险加固等项目，财政总投入规模达25亿元以上，惠及更多城乡居民。在实施过程中，扎实推进制度化规范化建设；在投入保障上，以预算安排为主体，群众筹资和社会捐赠为补充；在项目实施上，补助类项目坚持管理一体化、平台一网联、对象一线实、发放一卡通、服务一站办，补偿类项目坚持保障适度、收支平衡、略有结余，工程类项目坚持公开公平公正，加快实施进度，确保工程质量，严格责任追究；在监督检查上，实行部门监管、群众监督、社会化核查相结合。着力促进民生工程与社会保障体系、社会事业发展的有效衔接，让广大群众在更高水平上共享改革发展成果。
　　努力扩大就业和社会保障。以增加就业岗位为核心，高度重视返乡农民工技能培训和就业扶持，积极组织开展用工招聘会等各类就业援助活动，切实关注高校毕业生、复转军人等重点人群就业，重点促进零就业家庭就业和下岗失业人员再就业，实现再就业1.21万人，就业困难人员就业2600人。 加强劳动仲裁和监察，构建和谐稳定的劳动关系。持续扩大社会保险覆盖面，继续调高企业退休人员养老金，强化社会保险费征缴和基金监管。稳步推行征地补偿准备金制度，切实解决好被征地农民就业、住房和社会保障等问题。扩大新型农村养老保险试点，完善农村困难群众、城镇低收入人群生产生活救助和保障制度，强力推进以五保户集中供养为主的农村养老机构建设，对五保对象实现应保尽保。关爱农村“留守儿童”和“空巢老人”，发展慈善、社会福利和老龄服务业，支持残疾人事业。
　　推进生态市建设。进一步强化节能减排目标管理责任制，扎实开展环保专项行动，继续抓好“十项节能攻坚”，推进公共机构节能。加快重点工业污染源治理进度，逐步提高市县污水处理厂运行负荷，实行雨污分流，扩大管网覆盖面。加强森林资源保护和利用，切实保障饮用水源安全，着力推进生态公益林、退耕还林、农田林网等生态工程，治理水土流失面积100平方公里。进一步提高百千万生态示范工程创建水平，强化农村环境综合整治，加大环境优美乡镇、生态村创建力度。扎实开展建设用地置换和土地整理，完成第二次土地调查和土地利用总规修编。
　　维护社会安全稳定。坚持安全发展，层层落实政府的监管责任和企业的主体责任，继续深入开展重点行业和领域安全专项整治。落实领导和工作责任，加强食品药品等市场监管和专项整治，让人民群众吃得放心、用得放心。认真落实信访和维稳工作责任制，健全矛盾纠纷排查调处长效机制，坚持领导带头接访和带案下访，进一步畅通群众诉求渠道。完善应急管理体系，进一步巩固公安三基工程建设成果，继续充实和加强基层一线警力，加强公安信息化建设，构建平安六安。支持驻地部队建设，大力加强国防后备力量和民兵应急分队建设，积极稳妥在民营企业、大专院校开展国防动员和组建武装部，深入开展军民共建、创建双拥模范城活动。继续推进宗教工作“五抓”措施的落实，发挥宗教在促进社会和谐方面的作用，促进民族团结进步。
　　（七）统筹发展社会事业，推动社会全面进步。在加快经济发展的基础上，把社会事业发展摆到更加突出的位置，使经济和社会事业发展相互促进、相得益彰，更加协调一致。
　　加快社会事业发展。坚持教育优先，推进教育均衡，扩大职业教育规模，加快普及高中教育，重视幼儿教育，全面提升教育质量。加快新一轮中小学布局调整、农村寄宿制学校建设和城区义务教育学校改扩建，开展中小学标准化建设。深化医药卫生体制改革，加强公共卫生、农村卫生和社区卫生体系规范化建设，实施市中医院、二院综合病房大楼和市肿瘤医院等项目建设；完善新农合制度，推进爱国卫生运动，支持六安卫校争创安徽卫生职业学院。全面加强人口和计划生育工作，稳定低生育水平，扎实推进“七到位两规范”各项措施的落实，健全“村为主”工作机制，综合治理出生人口性别比偏高问题，完成计划生育技术服务机构规范化建设。广泛开展全民健身运动，提升竞技体育水平。创新人才工作机制，全面加强各类人才队伍建设。支持工会、共青团、妇联等人民团体独立自主地开展工作，推进外事、侨务和对台工作，做好统计、人防、地震、气象、地方志、档案等工作。
　　促进文化发展繁荣。推进文化资源信息共享、广播电视村村通、数字电视、手机电视、电影2131、送戏下乡等文化惠民工程。以庆祝建国60周年为主题，积极开展群众性文艺创作和演出活动。大力发展文化产业，推进文化产业规划编制和重大文化产业项目带动战略。积极争取启动市图书馆、文化馆、文化大世界、报业大厦等项目建设。实施文化展示工程，注重文化遗产保护利用，加快文化旅游开发，推进文化事业单位体制改革，加强新闻出版、演出娱乐、网吧等文化市场的经常性管理。
　　加强民主法制和思想道德建设。深入开展“五五”普法，加强法律援助，抓好社区矫正试点，推进村务公开和民主管理示范创建活动。把建设社会主义核心价值体系融入国民教育和精神文明建设全过程，大力倡导社会主义荣辱观，深入开展社会公德、职业道德、家庭美德教育，加强未成年人思想道德建设。坚持正确舆论导向，充分发挥新闻媒体作用。广泛开展和谐城市、和谐村镇、和谐社区、和谐家庭创建活动，努力形成社会整体和谐的良好局面。

**三、求真务实，全面加强政府自身建设**

　　应对各种挑战，做好2009年各项工作，我们必须坚持以科学发展观为统领，以更加昂扬的斗志、更加务实的作风、更加有为的工作，不断开创跨越崛起的新局面。
　　继续解放思想。把解放思想作为贯彻落实科学发展观的前提和基础，贯穿于政府工作始终。通过解放思想，大力弘扬锐意改革、敢为人先的精神，着力增强迎难而上的锐气、破解难题的勇气。通过解放思想，用创新的思维和办法应对新情况，解决新问题。通过解放思想，切实增强大局观念和全局意识，充分调动全市上下推动发展、加速崛起的积极性和创造性。各级政府要带头解放思想，开明开放，敢闯敢试，做改革的促进派、创新的实践者。
　　遵循科学发展。深入开展学习实践科学发展观活动，以科学发展观武装头脑、指导实践、推动工作。更加注重发展质量，切实抓好全局性、牵动性、导向性的大事，致力于加强薄弱、夯实基础、事关长远的工作；更加注重城乡统筹、区域协调，努力形成以城带乡、以乡促城、城乡互动、竞相发展的良好局面；更加注重以人为本、关注民生，集中精力解决人民群众最关心、最直接、最现实的利益问题。各级政府一定要自觉践行科学发展观，切实提高领导科学发展和驾驭复杂局面的能力。
　　推进依法行政。深入贯彻《国务院关于加强市、县政府依法行政的决定》，牢固树立法治观念，严格规范行政行为。坚持问政于民、问需于民、问计于民，对重大政策、重大项目安排和大额度资金使用等决策，深入调查研究，广泛听取意见，集体讨论决定。坚决贯彻落实市委决策部署，自觉接受人大法律监督和政协民主监督，认真办理人大议案和政协提案，广泛听取各民主党派、工商联、无党派人士及各人民团体的意见，自觉接受社会公众和新闻舆论监督。支持监察、审计等部门依法独立履行监督职责。
　　着力改进作风。积极倡导和培育敢于负责、一抓到底的作风，以抓铁有痕的精神，推动各项工作的落实。对重大决策和部署，对关键环节和重点难点问题，逐项分解，细化量化，落实到人，做到明责任，抓到位，见效果。进一步优化发展环境，切实提高行政效能，坚决摆脱文山会海，严格控制各类检查评比，集中精力、群策群力谋大事、谋发展，抓具体、抓落实。各级政府要牢固树立艰苦奋斗、勤俭节约的思想，带头压缩支出，集中更多资金用于保增长、保重点、保民生。
　　坚持廉洁从政。认真贯彻落实《建立健全惩治和预防腐败体系2008－2012年工作规划》，严格执行党风廉政建设责任制和领导干部廉洁自律的各项规定，加强领导干部修养，健全干部任期经济责任审计等制度。围绕“管人、管权、管钱、管地”等，进一步巩固反腐倡廉制度建设推进年活动成果，完善和规范建设工程、国土资源交易、产权交易、政府采购、民生工程等招投标和履约监督管理及资金管理，建立健全决策权、执行权、监督权相互制约又相互协调的权力运行机制。继续抓好专项治理，坚决纠正损害群众利益的不正之风，确保政事清明、政风端正。
　　各位代表！全市人民共同努力所取得的成就让我们站在了一个新的起点，700万人民加快发展的强烈愿望激励着我们实现新的跨越。我们坚信，任何困难都改变不了皖西儿女创造美好生活的强烈愿望，任何挑战都阻挡不了老区人民加速跨越发展的坚定步伐！让我们在中共六安市委的坚强领导下，高举科学发展的大旗，同心同德，开拓进取，为全面建设小康六安的伟大目标而努力奋斗！
